# Influence of Familial Inflammatory Bowel Disease History on the Use of Immunosuppressants, Biological Agents and Surgery in Patients with Pediatric-Onset of the Disease in the Era of Biological Therapies. Results from the ENEIDA Registry

**DOI:** 10.3390/jcm14103352

**Published:** 2025-05-12

**Authors:** Carlos González-Muñoza, Antonio Giordano, Elena Ricart, Pilar Nos, Eva Iglesias, Javier P. Gisbert, Santiago García-López, Francisco Mesonero, Isabel Pascual, Carlos Tardillo, Montserrat Rivero, Sabino Riestra, Míriam Mañosa, Yamile Zabana, Fernando Gomollón, Xavier Calvet, Mariana Fe García-Sepulcre, Ana Gutiérrez, Jose Lázaro Pérez-Calle, Mónica Sierra-Ausín, Fernando Bermejo, Lara Arias, Manuel Barreiro-de Acosta, Jesús Barrio, Rufo Lorente, Jordi Guardiola, Pilar Varela, Ángel Ponferrada-Díaz, Ignacio Marín-Jiménez, Cristina Martínez Pascual, Esther Garcia-Planella, Eugeni Domènech

**Affiliations:** 1 Gastroenterology Department, Hospital Santa Creu i Sant Pau, 08025 Barcelona, Spain; 2 Departament de Medicina, Universitat Autònoma de Barcelona, 08193 Cerdanyola del Vallès, Spain; 3 Gastroenterology Department, Hospital Clínic, 08036 Barcelona, Spain; 4 Institut d’Investigacions Biomèdiques August Pi i Sunyer (IDIBAPS), 08036 Barcelona, Spain; 5 Centro de Investigación Biomédica en RED (CIBEREHD), 28029 Madrid, Spain; 6 Gastroenterology Department, Hospital Uniersitari i Politècnic La fe, 46026 València, Spain; 7 II-S La Fe, 46026 Valencia, Spain; 8 Gastroenterology Department, Hospital Reina Sofía, 14004 Córdoba, Spain; 9 IMIBIC, 14004 Córdoba, Spain; 10 Gastroenterology Department, Hospital Universitario de La Princesa, 28006 Madrid, Spain; 11 Instituto de Investigación Sanitaria Princesa (IIS-Princesa), 28006 Madrid, Spain; 12 Universidad Autónoma de Madrid (UAM), 28049 Madrid, Spain; 13 Gastroenterology Department, Hospital Universitario Miguel Servet, 50009 Zaragoza, Spain; 14 Instituto de Investigación Sanitaria de Aragón (IIS), 50009 Zaragoza, Spain; 15 Gastroenterology Department, Hospital Universitario Ramón y Cajal, 28034 Madrid, Spain; 16 Gastroenterology Department, Hospital Clínico de Valencia, 46010 Valencia, Spain; 17 Gastroenterology Department, Hospital Universitario Nuestra Sra. De la Candelaria, 38010 Santa Cruz de Tenerife, Spain; 18 Grupo de Investigación Clínica y Traslacional en Enfermedades Digestivas, Instituto de Investigación Valdecilla (IDIVAL), 39011 Santander, Spain; 19 Hospital Universitario Marqués de Valdecilla, 39008 Santander, Spain; 20 Gastroenterology Department, Hospital Universitario Central de Asturias, 33011 Oviedo, Spain; 21 Instituto de Investigación Sanitaria del Principado de Asturias (ISPA), 33011 Oviedo, Spain; 22 Gastroenterology Department, Hospital Universitari Germans Trias i Pujol, 08916 Badalona, Spain; 23 Gastroenterology Department, Hospital Universitari Mútua Terrassa, 08221 Terrassa, Spain; 24 University of Barcelona, 08007 Barcelona, Spain; 25 Gastroenterology Department, Hospital Clínico Lozano Blesa, 50009 Zaragoza, Spain; 26 Gastroenterology Department, Parc Taulí, Hospital Universitari, 08208 Sabadell, Spain; 27 Institut d’Investigació i Innovació Parc Taulí, 08208 Sabadell, Spain; 28 Gastroenterology Department, Hospital General Universitario de Elche, 03203 Elche, Spain; 29 Gastroenterology Department, Hospital General Universitario Dr Balmis, ISABIAL, 03010 Alicante, Spain; 30 Gastroenterology Department, Hospital Universitario Fundación de Alcorcón, 28922 Alcorcón, Spain; 31 Gastroenterology Department, Complejo Asistencial Universitario de León, 24008 León, Spain; 32 Gastroenterology Department, Hospital de Fuenlabrada, 28942 Fuenlabrada, Spain; 33 Gastroenterology Department, Hospital Universitario de Burgos, 09006 Burgos, Spain; 34 Gastroenterology Department, Hospital Clínico Universitario de Santiago de Compostela, 15706 Santiago Compostela, Spain; 35 Gastroenterology Department, Hospital Río Hortega, 47012 Valladolid, Spain; 36 Gastroenterology Department, Hospital General Universitario de Ciudad Real, 13005 Ciudad Real, Spain; 37 Gastroenterology Department, Hospital Universitari Bellvitge, 08907 L’Hospitalet de Llobregat, Spain; 38 Gastroenterology Department, Hospital Universitario de Cabueñes, 33394 Gijón, Spain; 39 Gastroenterology Department, Hospital Universitario Infanta Leonor, 28031 Madrid, Spain; 40 IiSGM, 28009 Madrid, Spain; 41 Gastroenterology Department, Hospital Gregorio Marañón, 28007 Madrid, Spain; 42 Medicine Faculty, Complutense University Madrid, 28040 Madrid, Spain; 43 Gastroenterology Department, Hospital Clínico Universitario Virgen de la Arrixaca, 30120 El Palmar, Spain

**Keywords:** pediatric, familial history, inflammatory bowel disease, surgery, biologicals

## Abstract

**Background:** Pediatric-onset familial inflammatory bowel disease (IBD) may differ from sporadic pediatric-onset IBD in its genetic and environmental background and may have distinct clinical and therapeutic implications. **Objective:** To evaluate the influence of a positive family history of IBD on the use of medical therapies and surgical interventions in adult patients with pediatric-onset IBD. **Methods:** Retrospective case–control study using the Spanish ENEIDA registry, including adults diagnosed with pediatric-onset IBD since 2006. Familial forms (FFs) (defined by a first-degree relative with IBD) and sporadic forms (SF) (with no relatives of any grade with IBD) were matched 1:4 by type of IBD, sex, age at IBD diagnosis, disease location, disease pattern, development of perianal disease and smoking status at diagnosis. The study outcomes were the use of immunomodulators, biological therapies, intestinal surgery, and perianal surgery during follow-up. **Results**: Six-hundred and fifty-five Crohn’s disease (CD) (131 FF) and 440 ulcerative colitis (UC) (88 FF) patients were included. Immunomodulators, biological therapy, and intestinal surgery were used evenly among FF and SF patients for both UC and CD. However, a higher requirement for perianal surgery among FF-CD patients (18.3% vs. 10.5%, *p* = 0.014), together with a shorter time to perianal surgery (11 vs. 20 months, log-rank *p* = 0.004), was observed. **Conclusions:** Patients with FF of pediatric-onset IBD do not exhibit an increased use of immunomodulators, biological agents, or intestinal surgery, but do exhibit a higher need for perianal surgery, as compared to patients with SF pediatric-onset IBD.

## 1. Introduction

Inflammatory bowel disease (IBD), including Crohn’s disease (CD) and ulcerative colitis (UC), shows a stable incidence across Western countries, with an increasing prevalence over recent decades [1]. These trends partially resemble those reported in the pediatric population, in which increasing incidence rates are still observed in some Western countries [1,2,3,4]. In addition, an increase in prevalence of 4.6% in Western populations [5] and an up to ten-fold increase in Eastern countries [6] have been reported. These trends are probably related to multiple environmental factors such as urbanization, industrialization, exposure to antibiotics during childhood, as well as dietary changes [7].

The etiology of IBD remains poorly understood, but current hypotheses pose an interaction between environmental and genetic factors [8,9]. Familial forms (FFs) of IBD represent the intersection of these two major factors, with some studies showing their association with pediatric-onset IBD [10,11], particularly in very-early onset forms of the disease [12,13]. Historically, pediatric-onset IBD was estimated to account for up to 25% of newly diagnosed cases of IBD [14,15]. However, more recent data suggest that this percentage may be lowered to approximately 8% in some populations [16,17]. Genetic factors may play a predominant role over environmental factors in pediatric-onset IBD. Likewise, a higher prevalence of certain genetic polymorphisms has been reported in patients with pediatric-onset IBD compared to adult-onset forms of the disease [18]. This genetic burden may explain, at least in part, some of the phenotypic differences observed in pediatric-onset IBD, such as higher rates of a familial history of IBD [19,20,21], male predominance [22], predominance of CD over UC [22], greater disease extent [21], a higher incidence of perianal disease [22], as well as an increased use of immunomodulators (IMM) and biological agents [22]. Even among patients with pediatric-onset IBD, those presenting before the age of six (very early-onset IBD) [23] show a higher rate of familial history of IBD [6], unclassified colitis [24,25], colonic location [6], and higher rates of infliximab discontinuation and surgery rates [26].

Some differences have been reported between pediatric-onset and familial forms of IBD when compared to sporadic forms (SF) of pediatric-onset IBD, including earlier age at clinical presentation and diagnosis [27], higher rates of reclassification of the IBD type [28], proximal progression (UC) [29], penetrating pattern (CD) [30] and higher enteral nutrition requirements [30]. Most of these studies were conducted before the widespread use of biological agents in pediatrics [29,31] and involved non-European populations [30] or single-center cohorts [32,33]. Studies comparing pediatric-onset FF and SF reported controversial results regarding surgical requirements, with no differences in the pre-biological era [27,29,31] but higher surgical requirements in FF in the biological era [34], particularly in UC. However, subsequent single-center and smaller studies with short follow-up periods found no differences [30,32,35,36].

Recently, our group compared FF and SF of adult-onset IBD patients observing similar phenotypes and showing no significant differences in medical or surgical management [37]. However, due to the previously reported phenotypic differences as well as a different therapeutic management [22], data on adult-onset IBD cannot be extrapolated to pediatric-onset IBD. The aim of this study is to evaluate the influence of a positive family history of IBD on the use of medical therapies and surgical interventions in adult patients with pediatric-onset IBD.

## 2. Materials and Methods

### 2.1. Study Design

This is an observational, retrospective, multicenter, case–control study. Patients were identified from the ENEIDA registry, which is a prospectively maintained registry set up in 2006, containing demographic, clinical, and treatment-related data of patients with IBD, promoted by the Spanish Working Group on Crohn’s Disease and Ulcerative Colitis (GETECCU) [38]. The registry was approved by the local Ethics Committees of all the participating centers, and all patients signed the informed consent form.

### 2.2. Study Population, Data Collection, and Definitions

The inclusion criteria were the following: (1) patients aged 17 years or younger at the time of IBD diagnosis; (2) Caucasian ethnicity and born in Spain; (3) IBD diagnosis made after December 2005 and followed up prospectively at the same center. Patients with indeterminate or unclassified IBD were excluded.

The ENEIDA registry includes the familial history of IBD and the degree of kinship with the index case. FF patients were defined as those with at least one first-degree relative diagnosed with IBD. SF patients were defined as those with no family members (of any degree) with IBD. Patients with a family history of IBD other than first-degree relatives were excluded from the study. Each patient with pediatric-onset FF of IBD was matched with four pediatric-onset SF patients using a propensity score (nearest value method, tolerance of 0.01) by type of disease, sex, age at diagnosis, disease location, disease pattern (according to the Montreal classification) [23], perianal disease at any time (CD), and smoking habit at IBD diagnosis.

Follow-up was defined as the time between IBD diagnosis and the last visit, loss to follow-up, or death, whichever occurred first.

Data recorded included demographic features, date of IBD diagnosis, age at diagnosis, smoking habit at diagnosis, IBD phenotype and location according to the maximum extent of the disease using the Montreal classification, perianal disease and extraintestinal manifestations, use and date of initiation of the first IMM, use and date of the first biological agent during follow-up, date of the first IBD-related abdominal and perianal surgery, and date of last appointment.

### 2.3. Statistical Analysis

The Kolmogorov–Smirnov test was used to assess the normality of the distribution of continuous variables. Variables with a normal distribution were expressed as mean and 95% confidence interval (CI) and compared using Student’s *t*-test. Variables with a non-normal distribution were expressed as median and interquartile range (IQR) and compared using the Mann–Whitney U test. Categorical variables are expressed as absolute values and frequencies, and the Chi-square or Fisher’s exact test was used for comparisons. In case of statistically significant differences between groups, a binary logistic regression analysis was used to measure the effect size. Patients with UC and CD were analyzed separately to assess whether there was a different impact on the management of the two diseases. Kaplan–Meier curves were used to evaluate survival time free of immunomodulators, biological agents, and surgery. The log-rank test was used to compare survival curves between FF and SF. *p*-Values < 0.05 were considered statistically significant.

## 3. Results

Among the 79,696 patients included in the ENEIDA registry at the time of data extraction (October 2024), 1693 (989 CD and 704 UC) met the selection criteria. After matching, 655 CD (131 FF and 524 SF) and 440 UC (88 FF and 352 SF) patients were included in the analysis (Table 1 and Table 2).

The median time of follow-up was 100 months (99.5 months [54–148] for CD and 102 [50.3–148] months for UC). Overall, compared to UC, a higher proportion of CD patients were males (63.7% vs. 43.2%, *p* < 0.0001), active smokers at diagnosis (6.3% vs. 2.3%, *p* = 0.0044), developed perianal disease along the disease course (30.1% vs. 4.6%, *p* < 0.0001), and changed the diagnosis of IBD type (3.5% vs. 1.1%, *p* = 0.026). Extraintestinal manifestations developed in 16.1% of patients during follow-up. Among UC patients, the most frequent extent was extensive UC (61%). In CD, the most frequent location was ileo-colonic (46.3%), and the most frequent disease behavior at the end of follow-up was the inflammatory pattern (79.4%). Regarding FF and SF, no differences were found in baseline characteristics.

### 3.1. Immunomodulators

**Figure 1:**
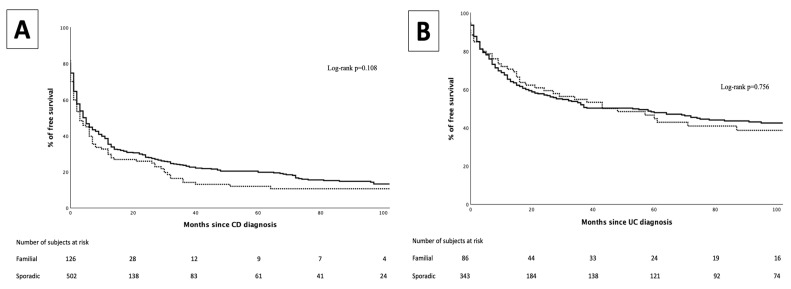
Immunosuppressant-free survival curve for familial forms (dotted line) and sporadic forms (continuous line) in Crohn’s disease (**A**) and in ulcerative colitis (**B**).

In the CD group, 537 patients (82%) were exposed to IMM. No differences were observed in the proportion of patients exposed to IMM between FF and SF (83.2% vs. 79.7%, *p* = 0.684), nor in the median time to IMM introduction (two months [0–7] vs. two months [0–12], *p* = 0.271) (Figure 1).

Of all UC patients, 239 (54.3%) were exposed to IMM. No differences were observed in the proportion of patients exposed to IMM between FF and SF (53.4% vs. 54.5%, *p* = 0.848), nor in the median time to IMM introduction (11 months [IQR 1–35] vs. 8 months [IQR 2–22], *p* = 0.641) (Figure 1).

### 3.2. Biological Therapy

**Figure 2:**
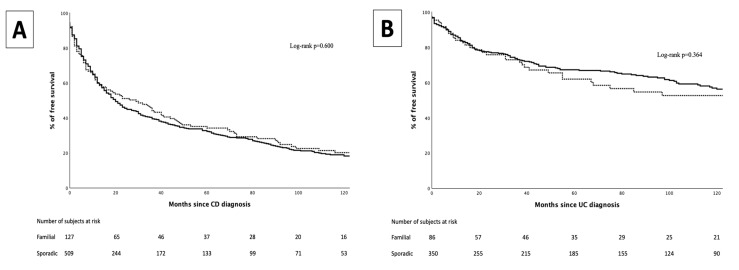
Biological-free survival curve for familial forms (dotted line) and sporadic forms (continuous line) in Crohn’s disease (**A**) and in ulcerative colitis (**B**).

In the CD group, 511 patients (78%) were exposed to at least one biological agent (46% adalimumab, 50.1% infliximab, 2.2% ustekinumab, 0.2% certolizumab, 0.2% vedolizumab, others 0.6%). No differences were observed between FF and SF in the type of biological agent used (*p* = 0.238). No differences were observed between FF and SF in the proportion of patients exposed to biological agents (78.2% vs. 77.1%, *p* = 0.777), or in the median time to the introduction of the first biological agent (12 months [IQR 3–45] vs. 12 months [IQR 4–36], *p* = 0.795) (Figure 2).

In the UC group, 182 patients (41.4%) were exposed to at least one biological agent (23,1% adalimumab, 66% infliximab, 7.7% golimumab, 3,3% vedolizumab). No differences were observed between FF and SF in the type of biological agent used (*p* = 0.934). No differences were observed between FF and SF in the proportion of patients exposed to biological agents (44.3% vs. 40.6%, *p* = 0.529), nor in the median time to the introduction of the first biological agent (27 months [IQR 7–67] vs. 19 months [IQR 7–56], *p* = 0.596) (Figure 2).

### 3.3. Intestinal Surgeries

**Figure 3:**
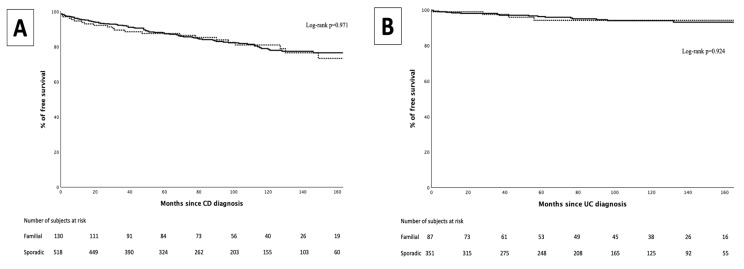
Intestinal resection-free survival curve for familial forms (dotted line) and sporadic forms (continuous line) in Crohn’s disease (**A**) and in ulcerative colitis (**B**).

In the CD group, 114 patients (17.4%) underwent intestinal resection. No differences were observed between FF and SF in the proportion of patients undergoing intestinal resection (17.6% vs. 17.4%, *p* = 0.959), nor in the median time to first intestinal surgery (30 months [IQR 6–90] vs. 42 months [11–78], *p* = 0.604) (Figure 3).

In the UC group, 22 patients (5%) underwent total or segmentary colectomies. No differences were observed between FF and SF in the proportion of patients that underwent total or segmentary colectomies (5.1% vs. 4.5%, *p* = 1), nor in the median time to colonic surgery (28 months [IQR 0–56] vs. 37 months [IQR 7–76], *p* = 0.412) (Figure 3).

Neither were the differences observed between FF and SF when only more aggressive forms of IBD were analyzed, such as extensive UC or ileal CD.

### 3.4. Perianal Surgeries

**Figure 4:**
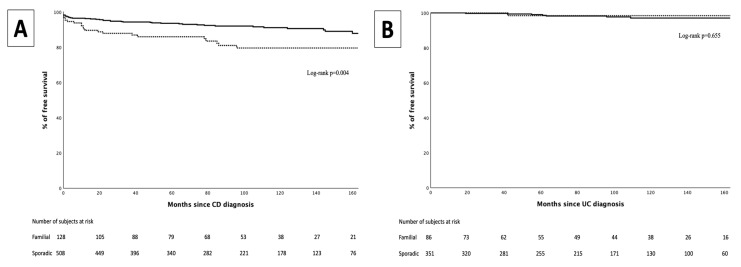
Perianal surgery-free survival curve for familial forms (dotted line) and Sporadic forms (continuous line) in Crohn’s disease (**A**) and in ulcerative colitis (**B**).

In the CD group, 79 patients (12.1%) required perianal surgery (abscess drainage, fistulotomy, seton placement). Between FF and SF, FF showed a higher perianal surgery requirement (18.3%) compared with SF (10.5%) (OR 1.91 [CI 95%: 1.13–3.23]; *p* = 0.014). Additionally, a significant difference was observed in the results of the time-to-event analysis (log-rank *p* = 0.004) (Figure 4).

In the UC group, nine patients (3.4%) required perianal surgery (abscess drainage, fistulotomy, seton placement). No differences were observed in the proportion of patients that required perianal surgery between FF and SF (2.3% vs. 2%, *p* = 0.655), or in the median time to perianal surgery (42 months [IQR 42–42] vs. 61 months [IQR 42–96], *p* = 0.500) (Figure 4).

## 4. Discussion

Genetic factors, which are believed to be implicated in familial and, particularly, in pediatric-onset forms of IBD, may play a pivotal role in both the phenotypic manifestations of diseases (which predominantly drive therapeutic strategies) and in response to drug therapy. To the best of our knowledge, this is the largest cohort of pediatric-onset IBD patients in which the impact (FF) on the use of IMM, biological agents, and surgery has been assessed. After long-term follow-up, we found no differences between FF and SF in the use of these treatments or the need for intestinal surgery, for both CD and UC, except for a higher rate of perianal surgeries in FF of CD.

Previous studies showed inconsistent results, likely due to methodological differences—particularly in how FF was defined. While some included only first-degree relatives [29,32,33,35,36], others considered up to third-degree relatives [25,28], and some lacked clear criteria [31,34], resulting in reported FF prevalence rates from 8.5% to 30%. To reduce variability and recall bias, we defined FF strictly as patients with first-degree relatives affected by IBD, excluding second- or third-degree cases, consistent with our previous adult-onset IBD study [37]. To ensure genetic homogeneity, only Caucasian patients born in Spain were included.

Because certain phenotypic traits associated with FF—such as earlier onset [27] or more extensive disease [29]—may influence treatment, we matched FF and SF groups for potential confounders like age at diagnosis, disease pattern, extent, and smoking. As in other pediatric-onset cohorts [27], we found no significant differences in phenotypic features relevant to treatment, such as perianal disease or extraintestinal manifestations.

Our study stands out for having a longer follow-up period than previously published studies [24,27,28,29,30,31,33,34,35,36], enhancing the accuracy of our findings, particularly regarding certain events such as surgery that often take years to be required. Additionally, all our patients were diagnosed with IBD after 2005, when biological agents were already widely used, to ensure a homogeneous cohort with broad access to contemporary guideline-recommended treatments [39,40,41], reinforcing the applicability of our results to current clinical practice.

As with other European cohorts [42,43], we observed a high and prompt introduction of IMM, as recommended in current European pediatric guidelines in UC [39,40]. In CD, their use had been recommended for maintenance therapy after exclusive enteral nutrition or steroids, as well as in combination with anti-TNF drugs [41]. Our results are in line with a similar Greek study [27] and some other small-scale studies [44].

Several studies did not observe differences in the use of biological agents between FF and SF [27,30,33]. Conversely, some studies in non-Caucasian populations of adult-onset IBD suggested an increased exposure to biological agents among FF [45,46,47,48,49]. However, many of these studies had methodological limitations such as short follow-up in SF [48], a study population of Ashkenazi Jewish origin (with a high familial aggregation) [45], being small-sized, single-center studies [46,49,50], or from geographical areas with restricted access to biological drugs [46,47]. The fact that we did not find differences in the time to biological introduction is unsurprising in CD, given that active inflammation is usually the indication for starting these therapies as soon as possible.

Similarly, no differences were found regarding the rate of intestinal resections between FF and SF of pediatric-onset IBD in several studies [27,29,30,31,32,35,36]. Conversely, in a study derived from the American PediIBDC Database [34], patients with UC and a first-degree relative with UC had an almost two-fold increased risk of colectomy. In contrast to intestinal surgery, FF of pediatric-onset CD had an increased rate (OR 1.91) and earlier requirement (log-rank *p* = 0.004) of perianal surgeries in our study. Due to the propensity score used, this cannot be explained by differences in disease location or pattern, though there are some plausible explanations for our findings. First, some genetic polymorphisms share an increased susceptibility to developing FF of IBD [27,51] and perianal disease [51,52]. Second, some polymorphisms have been associated with a worse response of perianal disease to antibiotics [53], potentially leading to an increased need for perianal surgery. Unfortunately, genetic data were not available in our study.

Our study has several strengths, such as its sample size, a strict definition of FF and SF, the homogeneous genetic background of the cohort, the matching of FF and SF by means of the phenotypic features that may drive therapy, and being an incident cohort at the time biological agents were already widely used. We are also aware of some limitations of our study. First, the ENEIDA registry includes mostly adult patients with IBD, and pediatric-onset IBD is likely to be underrepresented in the registry while patients are still under the age of 18. However, once the transition to adult IBD units is completed, these patients are introduced into the registry retrospectively and followed up prospectively. That may explain the exceedingly low number of patients diagnosed before the age of six. In addition, given the genetically homogeneous background of the cohort, our results may not be generalizable to non-Caucasian cohorts. Finally, the lack of genetic polymorphism data may also limit our results.

In conclusion, FF of pediatric-onset IBD is not associated with an increased use of IMM, biological agents, and intestinal resections, but is associated with a higher risk of perianal surgery. Having a family history of IBD in children with IBD should not be a criterion for changing the treatment algorithm or for anticipating a worse prognosis.

## Figures and Tables

**Table 1:** Characteristics of patients with ulcerative colitis according to sporadic and familial forms. Data are expressed in absolute value (frequency) and median (IQR).

	Sporadic (*n* = 352)	Familial (*n* = 88)	*p*-Value
Male sex	151 (42.9)	39 (44.3)	0.810
Age at diagnosis (*years*)	14 (11–16)	14 (12–16)	0.829
Very early onset disease (0–5 *years*)	16 (4.5)	4 (4.6)	0.901
Follow-up time (*months*)	99.5 (55–148)	109 (39–150)	0.721
Active smoking at diagnosis	8 (2.3)	2 (2.3)	0.777
Maximal disease extent			
Proctitis	44 (12)	10 (11)	
Left-sided	94 (27)	21 (24)	0.789
Extensive	214 (61)	57 (65)	
Perianal disease ever (fissure, fistulae, abscess)	10 (2.9)	5 (5.7)	0.196
Extraintestinal manifestations ever	51 (14.9)	8 (9.2)	0.227

**Table 2:** Characteristics of patients with Crohn’s disease according to sporadic and familial forms. Data are expressed in absolute value (frequency) and median (IQR).

	Sporadic (*n* = 524)	Familial (*n* = 131)	*p*-Value
Male sex	337 (64.3)	80 (61.1)	0.490
Age at diagnosis (*years*)	14 (12–15)	14 (12–16)	0.397
Very early onset disease (0–5 *years*)	18 (3.4)	6 (4.6)	0.131
Follow-up time (*months*)	98 (54–147)	102 (56–157)	0.558
Active smoking at diagnosis	32 (6.1)	9 (6.9)	0.578
Disease location *ileal/colonic/ileo-colonic/isolated upper-GI*	223/54/242/5 (43/10/46/1)	55/14/61/1 (42/11/46/1)	0.995
Disease behavior *inflammatory/stricturing/penetrating*	418/54/52 (80/10/10)	102/15/14 (78/11/11)	0.887
Upper GI involvement	149 (31.7)	38 (34.9)	0.525
Perianal disease ever	158 (30.2)	39 (29.8)	0.932

## Data Availability

The data underlying this article will be shared on reasonable request to the corresponding author.

## Abbreviations

The following abbreviations are used in this manuscript:

IBD	Inflammatory bowel disease
FF	Familial forms
SF	Sporadic forms
CD	Crohn’s disease
UC	Ulcerative colitis
IMM	Immunomodulators
GETECCU	Spanish Working Group on Crohn’s Disease and Ulcerative Colitis
